# Application of deep neural networks in automatized ventriculometry and segmentation of the aqueduct in pediatric hydrocephalus patients

**DOI:** 10.1007/s00234-025-03848-y

**Published:** 2025-11-27

**Authors:** Fabienne Kühne, Kilian Rüther, Christopher Güttler, Juliane C. Stöckel, Ulrich-Wilhelm Thomale, Anna Tietze, Andrea Dell’Orco

**Affiliations:** 1 Department of Neonatology, Charité - University Medicine Berlin, Berlin, Germany; 2 Institute of Neuroradiology, Charité – University Medicine Berlin, Berlin, Germany; 3 Department of Neurosurgery with Pediatric Neurosurgery, Charité – University Medicine Berlin, Berlin, Germany

**Keywords:** Pediatric hydrocephalus, Ventricular segmentation, NnU-Net, VParNet, Deep learning, MRI analysis

## Abstract

**Purpose:**

This study validated VParNet and nnU-Net for ventricular segmentation in pediatric hydrocephalus, a condition characterized by irregular and asymmetric ventricular shapes.

**Methods:**

Manual segmentation of 139 MRI scans (ages range 2.6–20.3 years) was performed for the four ventricles and the aqueduct. A five-fold cross-validation was conducted for both models. VParNet was tested with its original weights and after retraining on pediatric data. nnU-Net was extended to also segment the aqueduct. Performance was evaluated using the Dice Similarity Coefficient (DSC), Intraclass Correlation Coefficient (ICC), and Minimal Detectable Change (MDC).

**Results:**

VParNet preprocessing failed in 20.9% of cases, requiring subject exclusion. Both models showed good to excellent segmentation accuracy and reliability (DSC: 0.87–0.95; ICC: 0.81–1.0). Retraining VParNet improved DSC scores. MDC values (0.05–3.0) indicated high sensitivity for the lateral and third ventricles and acceptable sensitivity for the fourth ventricle. Aqueduct segmentation remained challenging (nnU-Net: DSC = 0.68; ICC = 0.81; MDC = 0.04).

**Conclusion:**

All tested models performed well in pediatric hydrocephalus segmentation, with no fundamental differences in overall performance. However, nnU-Net demonstrated key advantages due to its lack of preprocessing requirements, which allow the successful handling of even the most challenging subjects. These features make it easily implementable for clinical applications, providing fast and reliable ventricular segmentation and quantification.

**Supplementary Information:**

The online version contains supplementary material available at 10.1007/s00234-025-03848-y.

## Introduction

Hydrocephalus patients represent a major group in pediatric neurosurgery and neuroradiology. This condition can arise from various factors, including brain hemorrhage following prematurity, tumor diseases, or cerebral malformations [1]. Treatment for patients varies based on the underlying cause, including removing obstructions in the cerebrospinal fluid (CSF) pathways, creating a bypass through endoscopic third ventriculostomy (ETV), or diverting CSF using a ventricular shunt system. Patients subsequently receive routine clinical and imaging evaluations to monitor the size and shape of the ventricles to verify the consistent and reliable functioning of their shunt system or ETV [2]. Traditionally, ventricular size is evaluated through qualitative visual assessments and via basic manual linear measurements, such as the frontal-occipital horn ratio [3]. Given the complexity of the ventricular system, simple two-dimensional measurements may only capture a part of the ventricular size. More precise three-dimensional measurements are often impractically time-consuming for daily clinical use, making the automation of this process through artificial intelligence (AI) an appealing option [4]. Previously, artificial intelligence (AI)-based measurements of the ventricular system were proposed to detect shunt failure [5]. Moreover, in cases with external ventricular drainage, AI-measured CSF volume changes correlated strongly with the actually drained CSF [6]. To date, the application of AI in pediatric (neuro)radiology remains limited, primarily adopting deep learning models that have been established in adult populations in computer tomography (CT) [5–7] and magnetic resonance imaging (MRI) [8–10]. 

The objective of this study is to train and validate two AI-based tools, VParNet [4] and nnU-Net [^11^], for reliable and accurate automatized measurements of the ventricular system. A recent meta-analysis [9] reviewed several deep-learning models for ventricular segmentation, predominantly based on convolutional neural networks (CNNs) trained on T1w or T2w MRI, CT, or ultrasound images. Among open-source, MRI-based CNNs, VParNet showed the best performance and was originally developed for adults with normal-pressure hydrocephalus (NPH) [4, 9]. NPH typically results in symmetric enlargement of the ventricles, a condition that contrasts with the often asymmetric ventricles observed in pediatric hydrocephalus cases. While the VParNet approach yields reliable results in adult NPH, its applicability to the more complex pediatric ventricular systems, especially within the context of a developing brain, remains uncertain. Conversely, nnU-Net has demonstrated promising potential across various adult organ segmentation tasks and was previously employed in the segmentation of the whole ventricular system on CT images [12]. Its standout feature is the ability to automatically configure its architecture and training parameters to suit different imaging modalities and datasets, requiring no manual input by the user.

The hypotheses of our study are.

1. After retraining the application of VParNet, developed for ventricle segmentation in adult NPH patients, achieves comparable reliability and precision in pediatric hydrocephalus cases.
2. nnU-Net achieves results that are comparable in quality and reliability to the highly specialized VParNet.
3. nnU-Net successfully segments the aqueduct, a narrow channel connecting the third and fourth ventricle.

## Materials and methods

### Patients and MRI data

**Fig. 1:**
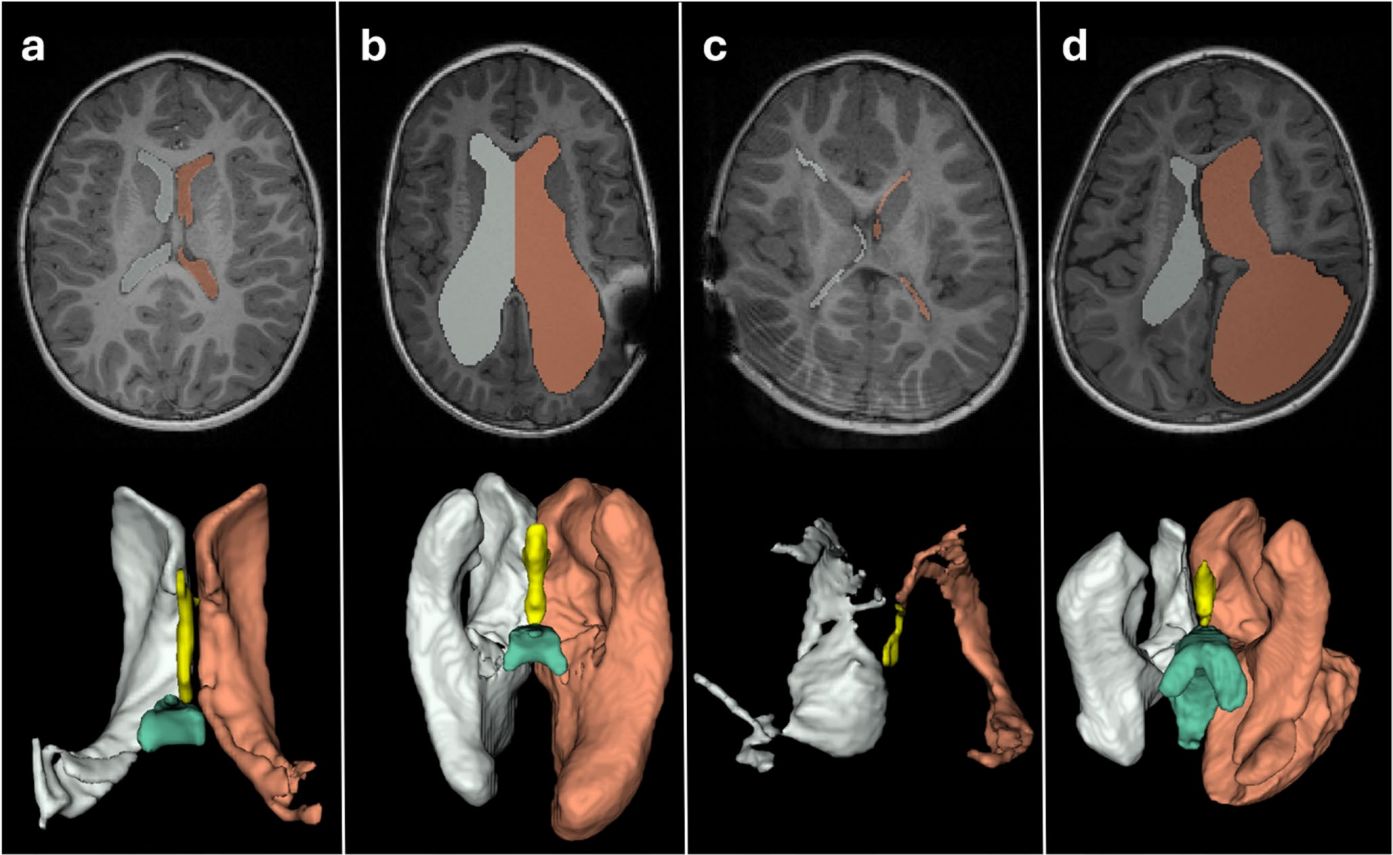
Examples for included subjects with **a** normal sized ventricles, **b** large ventricles and artefacts of shunt valve on the left side, **c** narrow ventricles and artefacts of shunt valves on the right side and motion artefacts, **d** irregular shaped large ventricles

We searched our Picture Archiving and Communication System (PACS; MERLIN Diagnostic Workcenter, Phönix-PACS GmbH, Freiburg, Germany) retrospectively from 2019 to 2021 to identify MRI studies of pediatric patients being evaluated for potential hydrocephalus or those undergoing monitoring following hydrocephalus treatment. MRI parameters are specified in Supp. Table 1. Special emphasis was placed on selecting cases with both asymmetrically and symmetrically enlarged ventricles, as well as instances of over- and underdrainage, meaning particularly large and very narrow ventricles, respectively (Fig. 1). Exclusion criteria encompassed movement artifacts, artifacts resulting from dental braces, the presence of tumors, recent hemorrhages, and significant cerebral malformations. Notably, studies displaying artifacts caused by shunt valves were not excluded.

**Table 1:** Description of the cohort

	subjects *n* (%)
*n*	139
Age 6.3 (IQR 2.3–11.4 years)	
Sexe	
male	78 (56.1%)
female	61 (43.9%)
etiology	
congenital (e.g. Chiari malformation, aqueductal stenosis)	86 (61.9%)
acquired	53 (38.1%)
posthemorrhagic	49 (35.3%)
post-infectious	3 (2.2%)
tumor	1 (0.8%)
ventricular size	
small	21 (15.1%)
normal	44 (31.7%)
wide	74 (53.2%)
asymmetric ventricular system	69 (39.6%)
symptoms at MRI acquisition	
yes	30 (21.6%)
no (MRI was a routine assessment)	109 (78.4%)
therapy	
shunt	91 (65.5%)
on right hemisphere	63 (69.2%)
on left hemisphere	21 (23.1%)
bilateral	4 (4.4%)
endoscopic third ventriculostomy	34 (24.5%)
no therapy	39 (28.1%)

This study was approved by the Charité Ethics Committee (EA2/282/21) and was conducted according to the guidelines of good clinical practice. Informed consent was waived in this retrospective study by local Ethic Committee.

### Manual outlining of ventricular systems

DICOM images were converted to the NIfTI format using dcm2niix [13]. The ventricular systems were manually delineated as regions of interest (ROI), using the paintbrush tool in ITK-SNAP [14]. The initial segmentations were carried out by a medical student (KR) trained by a pediatric neuroradiologist (AT). All segmentations were subsequently reviewed and, if necessary, refined by the neuroradiologist (AT). The lateral ventricles, the third and fourth ventricles, as well as the aqueduct, were identified and marked in different colors, while the choroid plexus and potential shunt catheters were deliberately excluded. The ventricular masks derived from this process were utilized as “ground truth” data.

### Validation and comparison of the deep-learning segmentation methods

Four segmentation methods were compared: (1) VParNet using the original weights released by Shao et al., [4] initially trained on adult NPH data, (2) VParNet after retraining on our pediatric cohort, (3) nnU-Net v2 (nnU-Net four classes), (4) nnU-Net v2 after adding the aqueduct as a fifth ROI (nnU-Net five classes). For 3. and 4. we opted for a full-resolution 3D architecture (3d_fullres nnU-Net option).

#### Preprocessing and quality control

VParNet: The original preprocessing pipeline from [4] was used, where the NIfTI images undergo a series of preprocessing steps including skull-stripping [15], N4 inhomogeneity correction [16], and coregistration to the MNI152 template [17]. Preprocessed MR images were visually inspected for systematic failures.

nnU-Net: Only the standard preconfigured automated preprocessing of nnU-Net was performed. To ensure consistency in neural network hyperparameters across all five folds of the cross-validation, the nnU-Net planning step was conducted only for the first data fold and exported to be used for the other four folds.

#### Cross validation

The dataset was split into five data folds and one of these folds was used as test dataset. The subjects in all the five training datasets for both methods were subjected to augmentation using TorchIO [18]. A composite transformation was applied to each subject, which consisted of a random flip angle between − 5° and 5° and a random elastic deformation of 0.05 magnitude. This operation resulted in a doubled number of subjects. Before performing the training, 20% of the subjects were randomly chosen to serve as validation.

For VParNet the original training parameters were used, kindly provided from the author of the original paper [4]. Training details can be found in Supp. Table 2.

**Table 2:** Median dice similarity coefficients (DSC), intraclass correlation coefficient (ICC), and minimal detectable change (MDC) were calculated to compare manual segmentations (ground truth) with four different segmentation methods: original VParNet, VParNet retrained on our pediatric cohort, nnU-Net four-class, and nnU-Net after incorporating the aqueduct as a fifth ROI (nnU-Net five-class)

ROI	VParNet – original weights	VParNet - retrained	nnU-Net four-classes	nnU-Net five-classes
DSC median [IQR]				
Left Lateral Ventricle	0.93 [0.88; 0.96]	0.92 [0.84; 0.95]	0.95 [0.90; 0.98]	0.95 [0.90; 0.97]
Right Lateral Ventricle	0.93 [0.87; 0.96]	0.92 [0.82; 0.96]	0.95 [0.92; 0.98]	0.95 [0.91; 0.98]
Third Ventricle	0.89 [0.76; 0.93]	0.87 [0.78; 0.91]	0.91 [0.86; 0.94]	0.91 [0.85; 0.94]
Fourth Ventricle	0.89 [0.80; 0.92]	0.91 [0.86; 0.93]	0.93 [0.89; 0.94]	0.93 [0.89; 0.95]
Aqueduct				0.68 [0.52; 0.78]
**ICC (LCI**,** UCI)**				
Left Lateral Ventricle	0.98 (0.98, 0.99)	0.99 (0.98, 0.99)	0.98 (0.98, 0.99)	0.99 (0.99, 1.00)
Right Lateral Ventricle	0.98 (0.98, 0.99)	1.00 (0.99, 1.00)	0.99 (0.99, 0.99)	0.99 (0.99, 1.00)
Third Ventricle	0.96 (0.93, 0.97)	0.98 (0.98, 0.99)	1.00 (0.99, 1.00)	0.99 (0.98, 0.99)
Fourth Ventricle	0.81 (0.68, 0.88)	0.92 (0.87, 0.94)	0.88 (0.83, 0.91)	0.86 (0.81, 0.90)
Aqueduct				0.81 (0.73, 0.87)
**MDC (cm³)**				
Left Lateral Ventricle	2.79	2.04	3.9	2.63
Right Lateral Ventricle	3.0	0.77	2.62	2.04
Third Ventricle	0.38	0.14	0.05	0.07
Fourth Ventricle	1.08	0.53	1.02	1.14
Aqueduct				0.04

IQR = interquartile range LCI = lower bound of the confidence interval, UCI = upper bound of the confidence interval, ROI = region of interest

Moreover, because the segmentation output from VParNet is in MNI152 ^18^ space, it was transformed back into native space.

### Statistical evaluation

The Dice Similarity Coefficient (DSC) [19] was chosen as the training loss function and metric to evaluate the segmentation performance of the two methods, as defined in Eq. 1.

Due to the violation of the homoscedasticity assumption, the effect of segmentation method on DSC was tested with Kruskal-Wallis test with Wilcoxon paired-samples signed-rank test employed as a post-hoc analysis. (α = 0.05). Bonferroni adjustments for p-values (indicated as p_adj_) were performed.

To evaluate the reliability of the volumes achieved via automatic segmentation, Intraclass Correlation Coefficient (ICC) and Minimal Detectable Change (MDC) [20] were calculated for each ROI and segmentation algorithm (Eqs. 2 and 3).

Data manipulation and calculation of DSC and volumes was performed with Python code with the following libraries: NiBabel [21], Numpy [22], Pandas [23]. Statistical evaluation was performed with R code and the libraries tidyverse [24], hrbrthemes [25], irr [26], rstatix [27]. 

## Results

### Patient characteristics

The cohort consisted of a total of 139 cranial MRIs from children (43.9% girls). The median age at the time of the MRI examination was 6.3 years (interquartile range, IQR 2.3–11.4 years; for details see Table 1).

### Manual outlining of the ventricular system

On average, the manual segmentation of each MRI examination required about half an hour. In some patients, the boundary between the lateral ventricles, known as the septum pellucidum, was indistinguishable due to agenesis or destruction. In these instances, a straight line drawn between the genu and splenium of the corpus callosum served as the delineation.

### Validation and comparison of the deep-learning segmentation methods

#### Quality control

Preprocessing failures were observed in 89 patients leading to the exclusion of 29 subjects (20.9%). VParNet descalping errors were observed in 17 cases (12.2%), with residual skull retained. Parts of the cortex were removed in 43 patients (30.9%) during descalping, however, sparing the ventricles, which was deemed negligible. More extensive tissue loss affecting both gray and white matter was seen in 27 patients (19.4%), extending toward the ventricles. Coregistration errors caused mis- segmentations in 2 cases (1.4%). For details see Supp. Figure 1. Overall, 29 subjects (20.9%) were excluded. In 2 cases of complete agenesis of the septum pellucidum and in one patient lacking the septum after surgery, the midline was defined as the straight line connecting the genu and splenium of the corpus callosum.

Because additional preprocessing is not part of nnU-Net, quality control was not needed.

#### Statistical evaluation

DSC, ICC, and MDC for the four comparisons, i.e., ground truth compared to the untrained VParNet, VParNet after retraining, nnU-Net four classes, and nnU-Net five classes, are reported in Table 2.

**Fig. 2:**
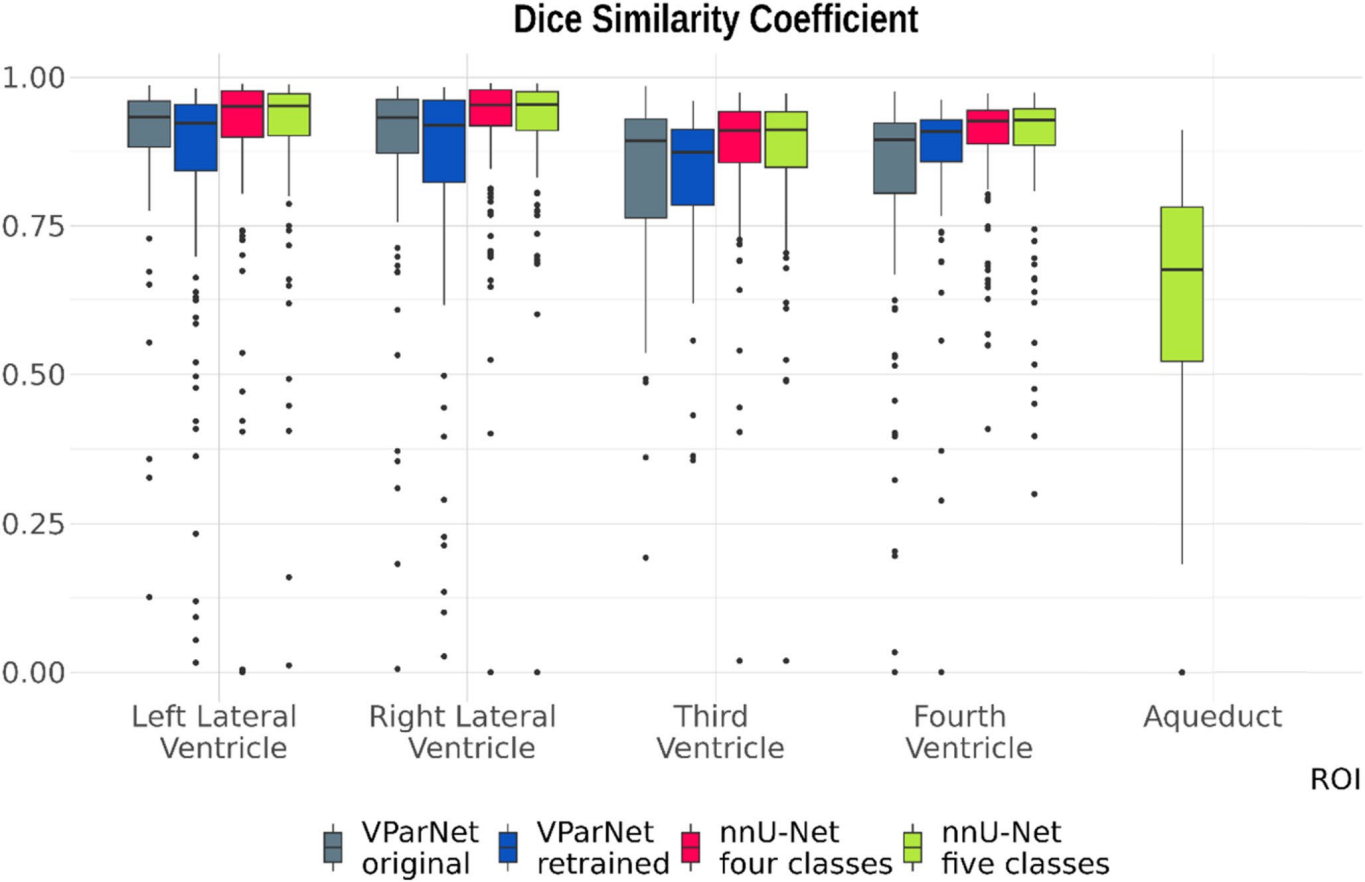
Boxplot of Dice similarity coefficients (DSC) for the four segmentation methods, respectively: untrained VParNet (grey), VParNet after retraining on our pediatric cohort (blue), nnU-Net four-classes (red), and nnU-Net five-classes (green)

**Fig. 3:**
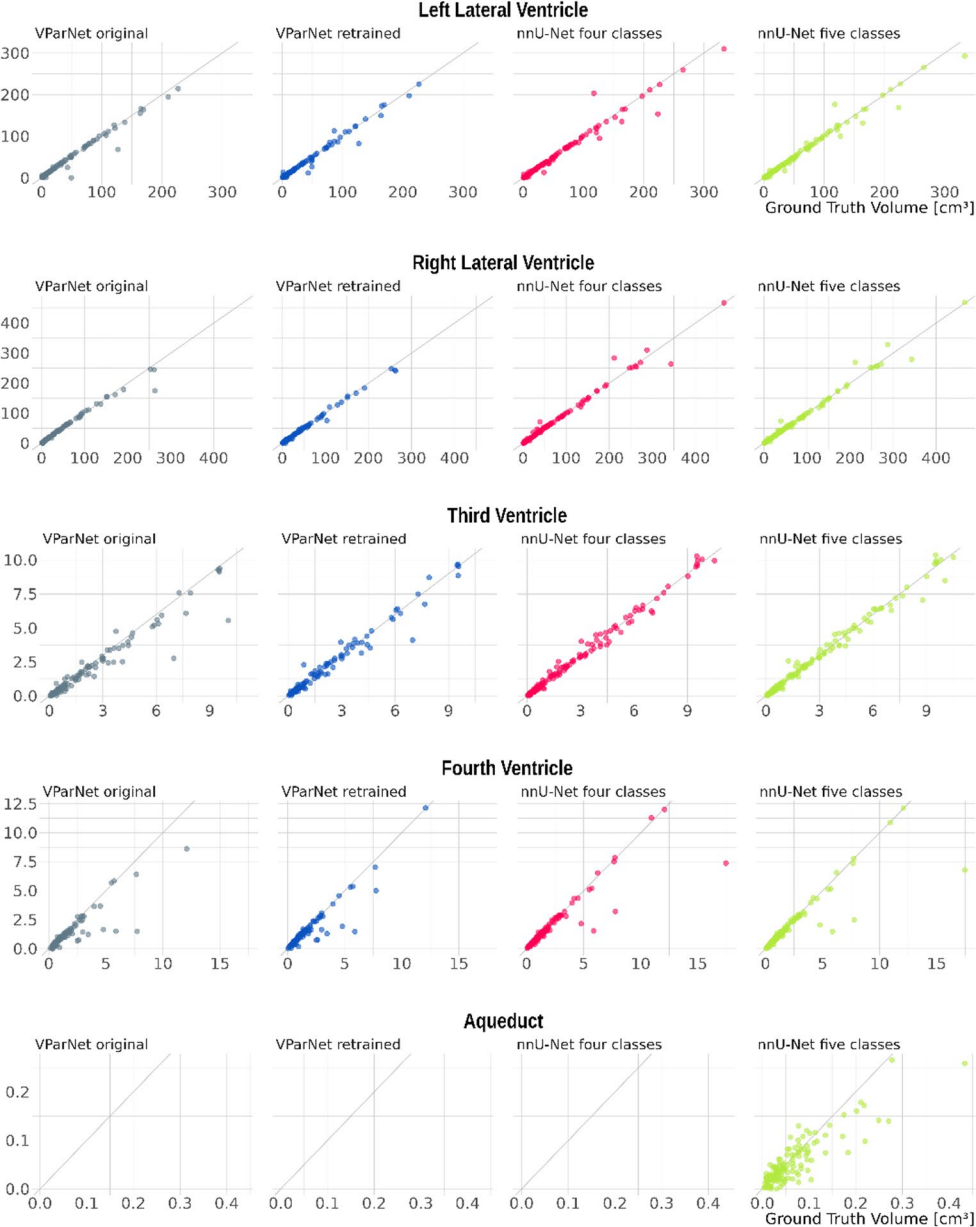
Correlation between the manually determined ground truth (x-axis) and automatically estimated volumes (y-axis). Grey: original VParNet; blue: retrained VParNet; red: nnU-Net four-class; nnU-Net five class

**Fig. 4:**
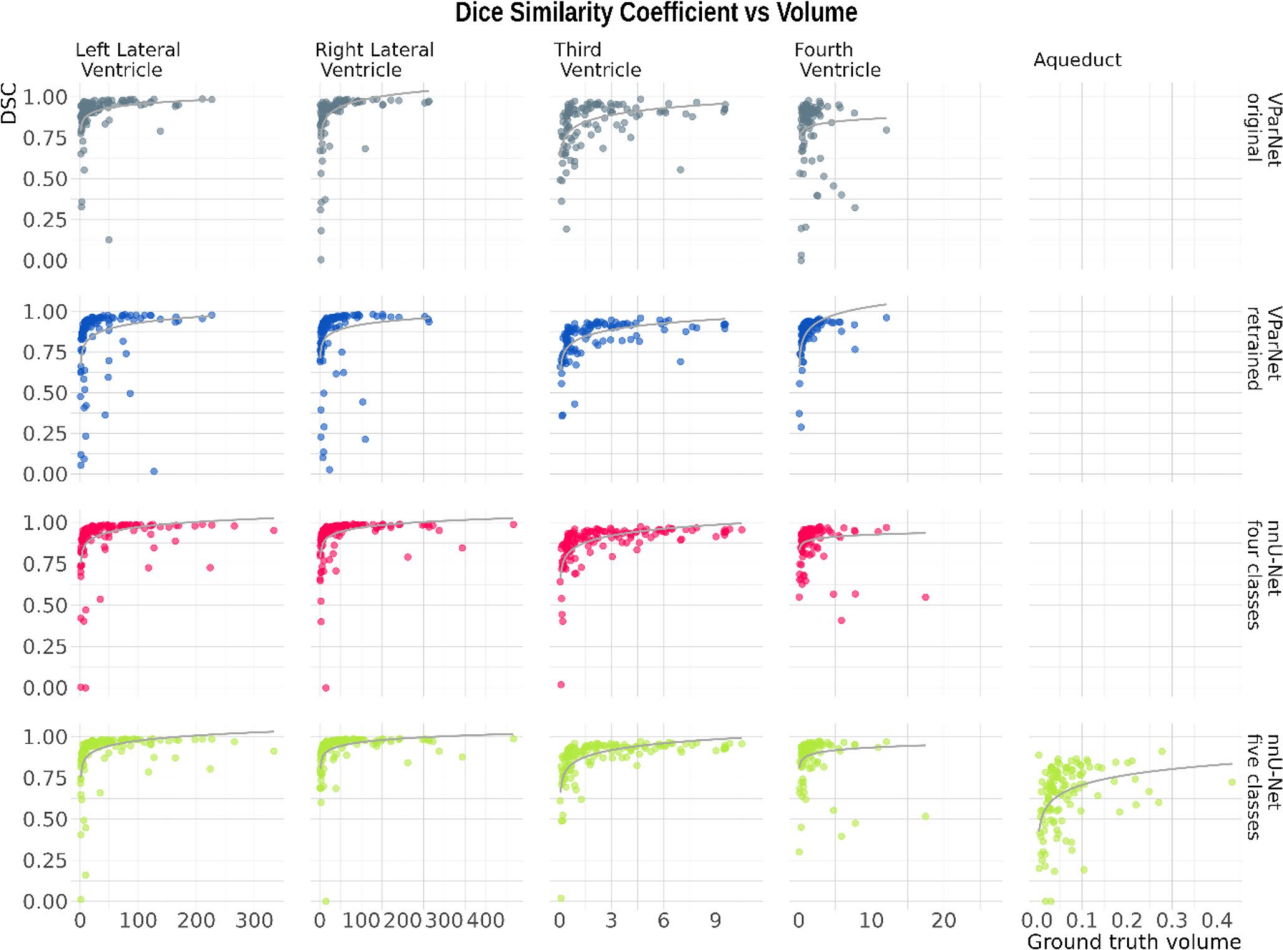
Distribution of dice similarity coefficients (DSC, y-axis) in relation to volume for the four ventricles and the aqueduct (x-axis) across the four models (grey: original VParNet; blue: retrained VParNet; red: nnU-Net four-class; green: nnU-Net five-class)

The segmentation quality of the lateral ventricles is excellent across all four algorithms, with median DSC values exceeding 0.92. For the third and fourth ventricles, both nnU-Net models also achieve excellent performance. The VParNet models perform very well, with DSC values of 0.89 (untrained) and 0.87 (retrained) for the third ventricle, and 0.89 (untrained) and 0.91 (retrained) for the fourth ventricle. However, segmentation of the aqueduct is noticeably less accurate, with a DSC of only 0.68. Figure 2 shows the distribution of DSC values for the four ventricles and the aqueduct, Fig. 3 the relationship between ground truth and automatically estimated volumes across the four algorithms. Figure 4 illustrates that the DSC is highly dependent on the volume of the segmented structure, which especially affects narrow ventricles and the aqueduct.

**Fig. 5:**
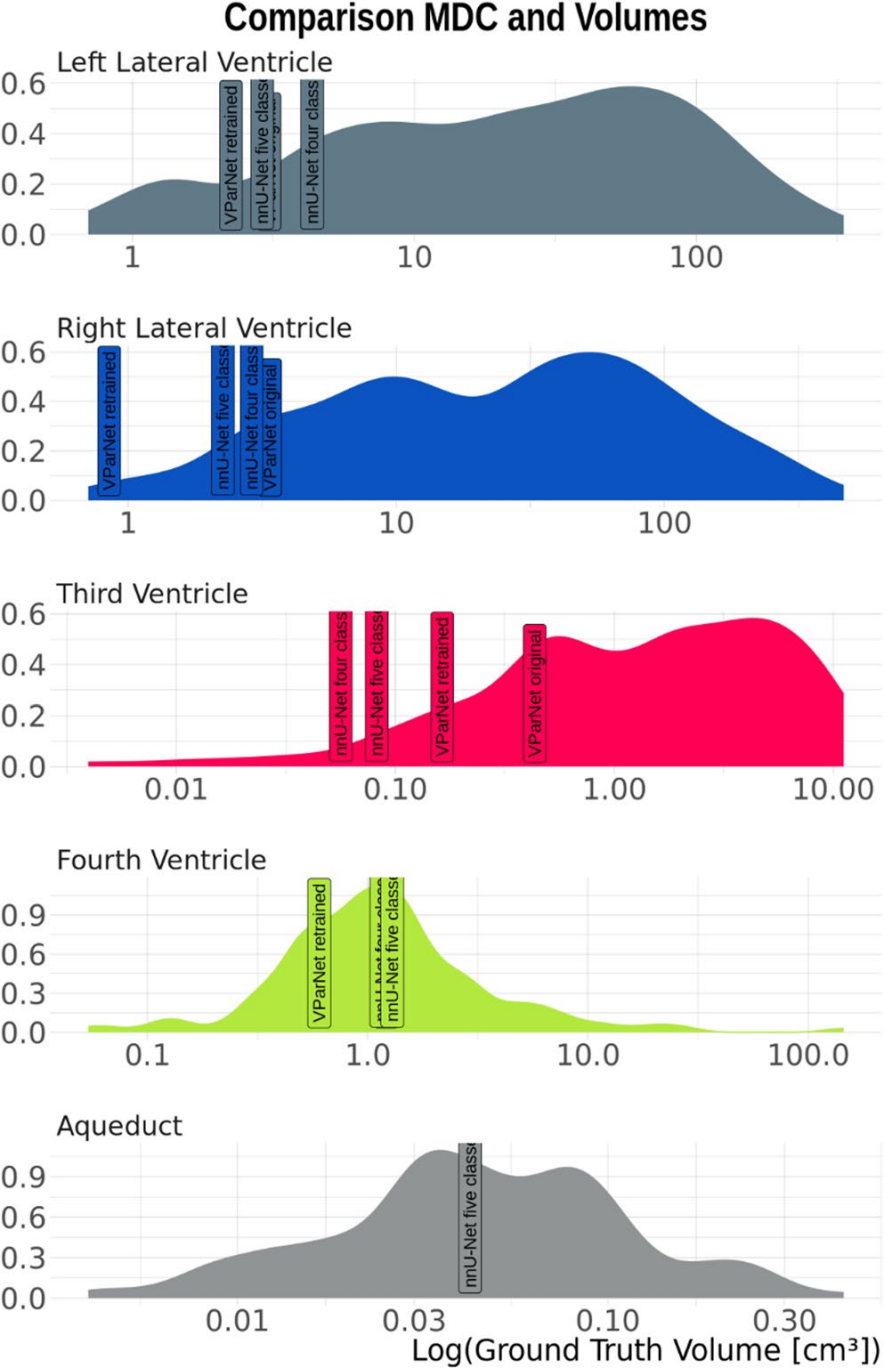
Minimal detectable change (MDC) values for the four models (original and retrained VParNet, nnU-Net four-class, nnU-Net five-class) plotted against the volume distribution of the four ventricles and the aqueduct (x-axis). MDC represents the sensitivity for detecting volume changes, with smaller values indicating more reliable measurements

There is a significant effect of the segmentation method on the right lateral (p_adj_ < 0.05) and fourth ventricle (p_adj_ < 0.05). Post-hoc analysis reveals no significant differences between the two nnU-Net segmentation methods and the two VParNet segmentation methods. In contrast, both the four-class and five-class nnU-Net methods significantly outperforms VParNet, with all comparisons yielding p_adj_ < 0.0001 for both original and retrained weights. Specifically, the four-class nnU-Net method outperforms VParNet for the right lateral ventricle (p_adj_ < 0.0001) and fourth ventricle (p_adj_ < 0.0001), regardless of whether original or retrained weights were used. Similarly, the five-class nnU-Net method also demonstrates superior performance over VParNet for both ventricles, with p_adj_ < 0.0001 for all comparisons. The ICC values indicate excellent agreement across algorithms for the lateral ventricles and the third ventricle (Table 2). The fourth ventricle also shows high reliability, with ICCs ranging from 0.81 (original VParNet) to 0.92 (retrained VParNet), while both nnU-Net models fall within this range. The ICC for the aqueduct is good, though not as high as for the ventricles. The MDC is smaller for all ventricles in VParNet when retrained, indicating that retraining improves the method’s sensitivity and reliability. Comparing VParNet and nnU-Net, there is no major difference between methods. Figure 5 shows the MDC values in relation to the volume distribution ROIs. The larger lateral ventricles tend to have lower MDC for all four algorithms, meaning that smaller volume changes can be detected more reliably, whereas smaller structures such as the fourth ventricle and the aqueduct show higher MDC relative to their size, indicating difficulty in detecting small changes accurately.

Although causing artifacts, shunt systems do not significantly affect DSC values for any ventricle across algorithms, except the aqueduct (*p* < 0.001). Additionally, ventricular asymmetry has no impact (*p* > 0.05).

## Discussion

The aim of this study was to validate neural networks for automated ventricular segmentation with the objective of monitoring pediatric hydrocephalus patients, who often present with highly irregular ventricular systems. The study explored two neural network-based methods: VParNet, previously used in adult NPH cases characterized by uniformly enlarged ventricles, and the nnU-Net, which has been applied to a range of medical image segmentation tasks. DSC values were high for both VParNet and nnU-Net, with nnU-Net outperforming VParNet (original/retrained) for all four ventricles. As expected, DSC values were slightly lower for the smaller third and fourth ventricles and the aqueduct. While high DSC values correlate with spatial and shape consistency, ICC and MDC primarily evaluate volume agreement. In a clinical setting, the reliable detection of volume changes is crucial, potentially indicating shunt blockage or CSF overdrainage. In our cohort, volume estimates showed good to excellent agreement across all algorithms, with minor differences when comparing MDC values between the original and retrained VParNet or ICC values between the third and fourth ventricles. However, MDC as a measure for an algorithm’s sensitivity to detect real volume changes was relatively higher for the fourth ventricle and the aqueduct. One possible explanation is that the exact anatomical boundaries of the aqueduct and fourth ventricle are poorly defined, contributing to variability. For instance, the caudal margin of the fourth ventricle at the CSF outflow tract may be unclear even to experts, leading to inconsistent volume estimates and, consequently, reduced sensitivity in detecting subtle volume changes. Studies reporting MDC values or longitudinal ventricular volume variations are rare. When comparing our MDC values with those reported by Nam et al.,^6^ who correlated drained CSF volumes with changes in CSF volumes derived using nnU-Net from CT, it becomes evident that our MDC values are substantially smaller than the drained CSF volumes reported in their work (approximately 5–15 mL).

Moreover, the results were not influenced by the presence of ventricular shunt systems that are often connected to a valve, which are typically causing considerable artifacts on MRI. In addition, we found no segmentation issues in patients with ETVs, where the floor of the third ventricle is partially absent. This was consistent across the two neural networks used in this study.

In our cohort, nnU-Net demonstrated significantly better DSC values for the lateral ventricles, but otherwise, there were no major performance differences, with none of the algorithm standing out overall. However, VParNet relies on a time-consuming and complex preprocessing step, which can introduce avoidable errors, a limitation not present in nnU-Net. These errors resulted in the exclusion of one-fifth of our dataset from further analysis. While manual correction or additional automation could address these issues, such an approach is impractical in clinical settings.

Automated ventricular segmentation can be a valuable clinical tool for detecting CSF pathway obstructions or absorption impairments, especially for longitudinal evaluation of patients with hemorrhage, infection, tumors, or hydrocephalus requiring surgical intervention. It improves treatment success evaluation by offering volumetric analysis rather than oversimplified linear measurements, particularly for irregular or asymmetric ventricles. It also enhances monitoring of shunt malfunctions and helps detect over- or underdrainage. Additionally, precise ventricular volume assessments can aid in personalizing intrathecal drug dosages for optimized treatment. Ventricular segmentation is a key part of several well-established segmentation pipelines, such as FreeSurfer [28] and FastSurfer [29]. While these atlas-based methods can also be applied, with a proper atlas, to neonates and infants, they can face significant challenges when encountering abnormal anatomy. Various approaches have been developed to optimize CSF and ventricle segmentation in hydrocephalic and anatomically abnormal pediatric brains. A study by Taha et al. reported the limited performance of two widely used whole-brain segmentation tools (Fastsurfer, QuickNAT) when applied solely to the lateral ventricles in a small pediatric cohort, with a mean DSC of 0.61 [^10^].

Russo et al. described a multiparametric approach that utilizes T1- and T2-weighted images to segment total brain and CSF volume in 23 subjects [30]. While this method is achieved excellent reliability on the investigated dataset, it does not allow separate volume assessment of individual ventricles. This limitation is critical because hydrocephalus is often localized to specific ventricular subspaces, such as in cases of aqueductal occlusion or adhesions within the ventricular system.

Largent et al. demonstrated excellent performance of a Bayesian U‑Net for ventricular segmentation in preterm infants with posthemorrhagic hydrocephalus, achieving very high Dice scores for the lateral ventricles (DSC ≈ 0.95). Their cohort, however, was restricted to neonates with relatively homogeneous pathology and focused primarily on the lateral ventricles compared to our larger and more heterogeneous pediatric cohort with hydrocephalus of varying etiologies and include segmentation of all four ventricles as well as the aqueduct [31]. 

Quon et al. used a 2D U-net to segment ventricular systems in 200 pediatric patients with posterior fossa tumors and hydrocephalus affecting the lateral and the third ventricles [32]. Patients with permanent shunt systems or ETV were excluded. Excellent performance with an overall DSC of 0.901 and strong correlation of ventricular volumes were obtained when comparing with manual segmentations. However, measures for individual ventricles are not reported in this study. Given the tumor localization, it is likely that the fourth ventricle was not included in the evaluation. Additionally, the ventricular shapes in this cohort may not be entirely comparable to those in our study. Extensive asymmetric tissue damage around the supratentorial ventricular system, common in our cases, may be underrepresented in Quon et al.‘s cohort, where ventricles are likely to be more evenly dilated, akin to the original NPH cohort assessed with VParNet [4]. 

Previous studies using nnU-Net for brain segmentation have not focused on ventricular system segmentation, particularly not in pediatric hydrocephalus patients. de Boer et al. [33] applied nnU-Net for ventricular segmentation in adult tumor patients, reporting similarly high DSC values (median 0.91). However, since individual ventricles were not evaluated separately, a direct comparison to our results is not possible. Consistent with our findings, Donnay et al. also reported excellent CSF segmentation using nnU-Net [34]. However, their study differs fundamentally in design, as it focused on adults with multiple sclerosis, utilizing multi-contrast MRI and without manual ground-truth comparisons.

An interesting approach to aqueduct segmentation was proposed by Tsou et al., [35] who utilized phase-contrast MRI. They compared two deep-learning architectures (U-Net and MultiResUNet) against manual segmentations, achieving excellent results (DSC > 0.92). However, a limitation of this approach is that it is exclusively tailored for aqueduct segmentation, meaning that other parts of the ventricular system cannot be segmented.

### Limitations and future improvements

To maximize the neural network’s ability to generalize to new, unseen data, we prioritized selecting a diverse and representative training dataset, intentionally including a wide range of ventricle shapes and sizes. While this approach aims to capture the complexity of real-world variability, we acknowledge that the inherent diversity of ventricle morphologies may still pose challenges for comprehensive representation, and therefore, some edge cases may not be fully accounted for. Relying exclusively on data from a single center with uniform examination parameters could adversely impact the model’s ability to generalize. Furthermore, the specific inclusion and exclusion criteria might also constrain the model’s broad applicability. However, to facilitate adaptation to different datasets, we provide an easily fine-tunable model. The considerable variability in the training data, encompassing MRI scans from patients with either extremely large or small ventricles and a broad age spectrum (from four months to 18 years) with varying signal ratios between gray and white matter. Furthermore, it would be valuable to include qualitative assessments of the ventricular system. For instance, tracking ventricular shape changes longitudinally, such as ballooning, bulging, or asymmetries of substructures, could give insights into underlying causes. Additionally, incorporating the segmentation of external CSF spaces would allow for a more accurate assessment of tissue atrophy and/or compression, providing a more comprehensive evaluation of the effects of pediatric hydrocephalus. To achieve this, to improve segmentation of small structures, e.g. the aqueduct, and to enhance generalization, multi-center data should be incorporated.

## Conclusion

In our cohort, high spatial agreement was found between manual segmentations and both VParNet- and nnU-Net-derived segmentations of the ventricular system. Volume estimates were also highly consistent across algorithms. While the sensitivity to detect real volume changes was high for larger structures like the lateral ventricles, it was lower for smaller structures, particularly the aqueduct. Although no fundamental differences were observed between the algorithms, VParNet has limitations in clinical applicability due to its time-consuming preprocessing and complex parameter optimization. In contrast, nnU-Net provides a fast, reliable, and easily implementable solution for automated ventricular volume measurement, even in highly distorted and asymmetric ventricular systems.

## Supplementary Information

Below is the link to the electronic supplementary material.

Supplementary file 1

## Data Availability

Due to data protection regularities, the MRI data is not openly available. The code used for this study is available under MIT license on GitHub (https://github.com/0rC0/VentriclesCNN), the pretrained models are released on OSF.io (https://doi.org/10.31219/osf.io/8em9u_v1). Moreover, in order to allow fine-tuning, we share a five-classes nnU-Net model trained on our whole dataset.

## Abbreviations

AI: artificial intelligence
CSF: cerebrospinal fluid
CT: computer tomography
DSC: Dice Similarity Coefficient
ETV: endoscopic third ventriculostomy
ICC: Intraclass Correlation Coefficient
IQR: interquartile range
LCI: lower bound of the confidence interval
MDC: Minimal Detectable Change
MRI: magnetic resonance imaging
NPH: normal pressure hydrocephalus
ROI: region of interest
SEM: standard error of measurement
UCI: upper bound of the confidence interval
