# Supplementary material for: Application of deep neural networks in automatized ventriculometry and segmentation of the aqueduct in pediatric hydrocephalus patients
Source: Neuroradiology. 2025 Nov 27;68(1):67–77. doi: 10.1007/s00234-025-03848-y (PMC12906524; doi:10.1007/s00234-025-03848-y)
Supplement: Supplementary file 1 [file 234_2025_3848_MOESM1_ESM.docx]

# Supplemental Material

## Tables

Supp. Table 1: MRI parameters

| **MRI parameter** | **details** |
| --- | --- |
| MRI Scanner | 3T Magnetom Skyra (Siemens Healthineers, Erlangen, Germany) |
| Head Coil | 64-element head coil (standard) |
|  | 8-element neonatal head coil (for infants under 2 months) |
| Series Included | 3D T1w (MPRAGE) |
| Voxel Size | 0.9 x 0.9 x 0.9 mm³ |
| Field of View (FoV) | 240 mm² |
| TR/TE/TI | 2,300 / 2.32 / 900 ms |
| Flip Angle | 8° |
| GRAPPA | 2 |
| Gadolinium Contrast | Not used |

Supp. Table 2: Training parameters for VParNet.

| **Parameter** | **Chosen adjustment** |
| --- | --- |
| Preprocessing Steps | -N4 Inhomogeneity Correction,  -Skull-Stripping  -Syn Registration to MNI 152 Template |
| Image Size | - Original Image Size After Preprocessing: 241 × 286 × 241 voxels  -Cropped Image Size: 192 × 256 × 192 voxels |
| Data Augmentation | Left-Right Flipping  Random Rotation  Elastic Deformation |
| Loss Function | 1- DSC |
| Training Parameters | Number of Epochs: 200epochs  Learning Rate: 1e-4  Batch Size: 1 |
| Images per Epoch | 160 (80 original images + 80 augmented images) |

## Figures


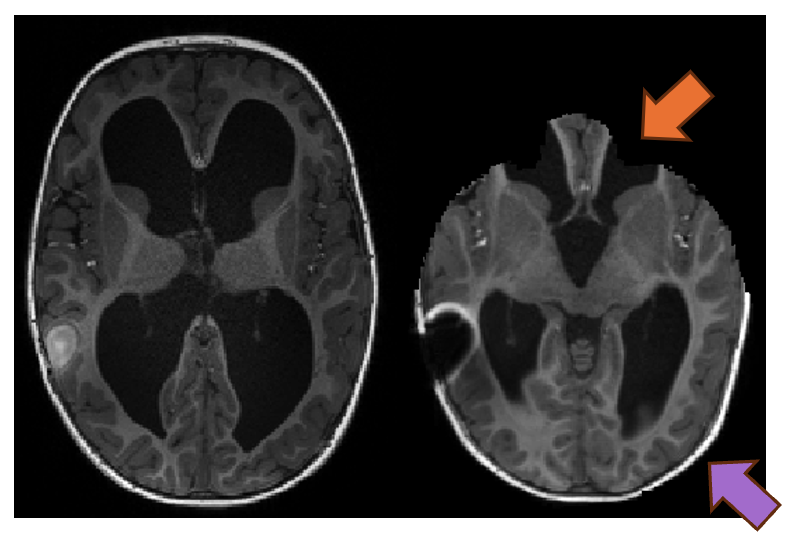


Supp. Figure 1: Axial MRI of a subject before (left) and after (right) faulty preprocessing of VParNet. The removal of the ventricular system is indicated with the orange arrow and the incomplete skull-stripping is indicated with a purple arrow.

## Equations

Equation 1

$$DSC =\frac{2 \times| A \cap B |}{| A | + | B |}$$

Calculation of DSC. Where ∣A∣ and ∣B∣ correspond to the number of elements in the sets being compared, and ∣A∩B∣ is the number of common elements between the sets. The DSC score ranges from between 0 to and 1, where 1 signifies a perfect match, and 0 indicates no overlap between the elements of sets A and B (14). The interpretation of the DSC score depends on the segmentation being assessed (14).

*Equation 2*

$$ICC = \frac{between-subject variance}{between-subject variance + within subject variance}$$

Calculation of ICC, which is used to assess the reliability. A higher ICC value indicates greater reliability (15). The ICC measures agreement between the ground truth and the respective segmentation algorithms with 0 for no and 1 for perfect agreement.

Equation 3

$$MDC = 1.96 \times\sqrt{2} \times SEM$$

with

$$SEM = \sqrt{within subject variance} = \sqrt{total variance \times(1 - ICC)}$$

Calculation of MDC and Standard Error of Measurement (SEM). MDC is the smallest change in a measurement that can be interpreted as a true difference, rather than a result of measurement error. A higher MDC value means that larger differences in measurements (15). The MDC is a measure for a true error that goes beyond random noise. In our study, the MDC is specified in mm^3^, in alignment to volumetric measurement. A lower MDC indicates more a sensitive method, capable of detecting smaller volume changes.
